# Sustainable Use of Agro-Industrial By-Products as Feed in Finishing Pigs

**DOI:** 10.3390/vetsci13010039

**Published:** 2026-01-02

**Authors:** Georgios Magklaras, Ioannis Skoufos, Eleftherios Bonos, Christos Zacharis, Konstantina Nikolaou, Evangelia Gouva, Ιlias Giannenas, Ioannis Giavasis, Athina Tzora

**Affiliations:** 1 Laboratory of Animal Science, Nutrition and Biotechnology, Department of Agriculture, School of Agriculture, University of Ioannina, Kostakioi Artas, 47100 Arta, Greece; gmag@uoi.gr (G.M.); ebonos@uoi.gr (E.B.); x.zaxaris@uoi.gr (C.Z.); 2 Laboratory of Animal Health, Food Hygiene and Quality, Department of Agriculture, School of Agriculture, University of Ioannina, Kostakioi Artas, 47100 Arta, Greece; knikolaou@uoi.gr (K.N.); egouva@uoi.gr (E.G.); tzora@uoi.gr (A.T.); 3 Laboratory of Nutrition, School of Veterinary Medicine, Faculty of Health Sciences, Aristotle University of Thessaloniki, 54124 Thessaloniki, Greece; igiannenas@vet.auth.gr; 4 Department of Food Science and Nutrition, School of Agricultural Sciences, Karditsa Campus, University of Thessaly, 43100 Karditsa, Greece; igiavasis@uth.gr

**Keywords:** swine, silage, fecal microbiota, fatty acids, meat characteristics

## Abstract

In modern animal production systems, the use of alternative feed sources is essential for reducing production costs, protecting natural resources, and improving meat quality. This study evaluated the impact of a unique silage—one produced by agro-industrial by-products—on the growth, meat quality, and health parameters of finishing pigs. Eighteen pigs (120 days old, 59.47 ± 0.85 kg) were randomly assigned to three dietary treatments containing 0% (A), 5% (B), or 10% (C) silage. After 60 days, blood samples were collected for hematological and biochemical analyses; this was followed by slaughter for the collection of muscle tissues (triceps brachii, external abdominal oblique) and intestinal digesta (ileum, cecum). Final body weight did not differ among groups (124.54 ± 1.51 kg), and meat composition (fat, protein, collagen, and ash) and pH were unaffected (*p* > 0.05). ALT levels were significantly lower in group C than in A (*p* = 0.030). In the ileum, *Enterobacteriaceae* and Enterococci populations decreased, whereas Lactobacilli increased in B and C (*p* ≤ 0.05). Cecal Enterococci were reduced in both B and C (*p* ≤ 0.01), with a tendency for increased Lactobacilli in B. Group C showed higher total phenolic content (*p* ≤ 0.05) and an improved ω-6/ω-3 ratio (15.09 vs. 17.54 in A). The TBARS values did not differ among treatments. *Campylobacter jejuni* counts were lower in treatment C, and no *Salmonella* or *Listeria monocytogenes* were detected in any samples. The meat color of the triceps brachii was redder in treatments B and C (*p* = 0.001). Overall, the innovative silage increased phenolic content, supported improvements in gut microbiota, and maintained meat quality, indicating its potential as a sustainable feed ingredient.

## Simple Summary

This study investigated the feasibility of incorporating a unique silage valorized from by-products of olive oil, winemaking, and feta cheese production as a sustainable dietary component for finishing pigs. The silage was added to pig diets at 0%, 5%, or 10% to evaluate its effects on growth, health, meat quality, and gut microflora. Overall, the pigs’ performance and meat composition, as well as most blood parameters, were not affected by the silage. The silage improved gut health by reducing harmful bacteria while the meat showed higher polyphenol content, a better fatty acid profile, and lower spoilage levels. Meat color was also influenced, with higher redness in silage-fed groups. No *Salmonella* or *Listeria monocytogenes* were detected in any samples. Overall, including this innovative silage in pig diets had no negative effects. Instead, it improved antioxidant properties, supported healthier gut microflora, and enhanced certain meat quality traits. Using agricultural by-products in this way may help reduce feed costs, lower environmental impact, and promote more sustainable pig production.

## 1. Introduction

In the modern era, livestock production has operated under escalating input costs and stringent sustainability constraints. Volatility in feed prices has intensified as climate change, competing land uses, and deforestation perturb global supplies of energy—and protein-rich feedstuffs. At the same time, European policy and consumer preferences are steering production toward healthier, more “sustainably produced” animal foods, and toward reduced on-farm antimicrobial inputs. The EU Farm-to-Fork agenda explicitly targets a 50% cut in sales of antimicrobials for farmed animals and aquaculture by 2030, while recent Eurobarometer work and allied surveys indicate that many consumers increasingly associate “healthy and sustainable” with low-pesticide, welfare-friendly, and lower-impact food chains [1,2,3].

In this context, researchers have intensified the search for the development and utilization of unconventional feed, primarily affordable and locally available feed resources that can partially replace imported soybean meal and cereals while also conferring benefits for animals and consumers. Southern Europe—and Greece in particular—produces large volumes of agro-industrial by-products (AIBPs) from three emblematic sectors: olive oil production, winemaking, and cheese manufacture. The Mediterranean basin generates approximately 30 million m^3^ of olive mill wastewater (OMW) annually. This effluent is rich in organic load and phenolics and, if mismanaged, is a significant pollutant, but is also a potential source of bioactive compounds [4,5]. Greece is among the world’s top olive oil producers, with around 430 thousand tons of olive oil per year processed in approximately 2800 mills—an industrial structure that underscores both the OMW burden and the valorization opportunity at the national level [6]. Depending on extraction technology, one ton of olives can yield up to approximately 1.6 m^3^ of OMW, again highlighting the magnitude of the stream that must be managed or valorized [7]. Wine by-products represent a second major AIBP resource. Across Europe, grape pomace (skins, seeds, and residual pulp) typically accounts for roughly 20–30% of processed grape mass, contributing millions of tons of fibrous, phenolic-rich biomass annually. Greek case studies and the data from Greece are consistent with respect to this range and document substantial volumes from national wine regions [8,9,10]. These lignocellulosic residues carry tannins and other polyphenols with antimicrobial and antioxidant properties but pose storage and handling challenges when used raw. Cheese processing ultimately produces large quantities of whey—the liquid fraction remaining after curd formation. Global whey output is commonly estimated at 145–200 million tons per year; unmanaged whey has very high biodegradability/total organic load numbers [high Biochemical Oxygen Demand/Chemical Oxygen Demand (BOD/COD)], making it an environmental liability but also a fermentable substrate in biorefineries and feed applications [11,12]. In Greece, where Feta, Protected Designation of Origin (PDO) dominates the cheese sector, 2021 production exceeded 130 thousand tons; given that approximately 10 L of whey are generated per kg of feta cheese, this single PDO category implies a production of more than 1.2–1.3 billion liters of whey annually—before accounting for other Greek cheeses—pointing to a substantial local feedstock for valorization (and, when de-proteinized, a source of solids useful in mixed silages) [13,14].

Despite their promise, the direct inclusion of these by-products in monogastric rations is technically challenging: high moisture, rapid spoilage, variable composition, anti-nutritional phenolics, and limited shelf life can compromise digestibility, palatability, and safety. Consequently, stabilization and “upgrading” via bioprocessing—ensiling, solid-state fermentation, and enzyme or microbial treatments—has become central to feed applications. Recent EU-Mediterranean work has shown that mixed-by-product silages (e.g., wheat straw, grape pomace, OMW, and cheese whey) can achieve desirable pH, lactic fermentation profiles, and preserved phenolics over 90 days, supporting their practicality as functional feed ingredients if well formulated [15,16,17,18].

Within this trajectory, a Greek research scientific group designed and optimized a novel mixed silage using locally abundant AIBPs—solids from olive mill wastewater and grape pomace, with the addition of de-proteinized feta cheese whey solids, arranged at approximately 60:20:20 (*w/w*) and supported by a cereal co-substrate to guide lactic fermentation [19]. In broiler chickens, dietary inclusion (5–10%) of this silage maintained performance levels and improved meat oxidative stability and lipid profile, indicating that the phenolic-fiber matrix can beneficially modulate product quality without penalizing growth [20].

Building on the poultry results, the same innovative silage was subsequently tested in pigs. In a pilot study, pigs received 0%, 5%, or 10% inclusion of the tested silage. Growth performance was maintained, while gut microbiota profiling revealed increases in beneficial taxa (e.g., *Bifidobacterium pseudolongum*) and reductions in potentially problematic *Streptococcus* spp., suggesting that the silage’s fermentable fiber and polyphenols reshaped the intestinal ecosystem in a potentially favorable way; carcass and meat-quality indices remained largely unaffected under the tested conditions [21,22]. Collectively, the evidence supports the hypothesis that silages formulated from agro-industrial by-products can partially substitute imported feed inputs while delivering antioxidant and microbiota-modulatory bioactives that promote host health, in alignment with EU antimicrobial-use reduction and sustainability directives.

European and Greek data indicate that (i) OMW streams in olive-producing regions (notably Greece) are large enough to justify feed-grade valorization pathways; (ii) grape pomace provides a seasonally abundant, phenolic-rich, fiber matrix compatible with ensiling; and (iii) cheese whey (or its de-proteinized solids) is both an environmental pressure point and a useful fermentable component in mixed silages [1,23,24]. When properly stabilized—most practically via ensiling and/or fermentation—these by-products can be blended into functional feeds for monogastrics such as pigs and poultry, with encouraging evidence for maintained performance; higher composition of antioxidant polyphenols, which may lead to improved oxidative stability of products during storage or further processing; and microbiome modulation [4,5,8,11,15,20,21,22]. Future work should focus on the finishing-pig production stage, refine inclusion levels beyond 10% where feasible, and incorporate techno-economic and life-cycle assessments to quantify cost savings and environmental co-benefits alongside animal health and product-quality outcomes [20,21,22].

The present study evaluated a new type of silage aimed at overcoming limitations in the utilization of locally generated agro-industrial by-products, specifically olive mill wastewater and grape pomace solids, as well as de-proteinized feta cheese waste solids. The silage formulation was established following optimization trials of the three components [19] and has been previously investigated in broiler chickens [20] and weaned pigs [22], yielding promising results in terms of health, productivity, and meat quality characteristics. To our knowledge, this is the first study evaluating this silage in finishing pigs. In doing so, we extend previous research conducted in weaned pigs by examining the effects of this application on growth performance, health indicators, and meat quality at the finishing stage, when feed consumption is highest and the valorization of agro-industrial by-products may provide considerable environmental and economic advantages.

## 2. Materials and Methods

### 2.1. Experimental Design and Treatments

All experimental procedures strictly followed the National Guidelines for Animal Trials (PD, 2013) [25] and received approval from the Department of Agriculture, University of Ioannina, Greece, through the University Research Committee (approval no. 61291/135/10.06.2020). The animal phase of the experiment was designed in compliance with the welfare considerations outlined in the Good Farming Practice Guidelines (Directive 2010/63/EC; Commission Recommendation 2007/526/EC). Throughout the experimental period, farming conditions and animal health were continuously monitored under veterinary and animal scientist supervision; all were members of the Department of Agriculture, University of Ioannina.

Animals were eighteen terminal crossbred pigs derived from Large White–Landrace maternal lines and Duroc paternal genetics, aged 120 days and obtained from an intensive pig production facility located near the university campus. Pigs were individually ear-tagged and randomly assigned to one of three dietary treatments: Control (0% silage), 5% silage, or 10% silage inclusion. The bioactive innovative silage tested as a feed additive in this trial was previously created following a novel multi-criteria mathematical optimization of the composition of 67 different recipes, as described by Petrotos et al. [19]. After fermentation of olive mill wastewater solids, grape pomace solids, and de-proteinized feta cheese whey solids, the optimal mixing ratio was chosen, and a novel silage was produced by establishing specific characteristics: low pH value (pH = 4.37), higher lactic acid content (total acidity = 2.52 expressed as lactic acid), higher lactic acid bacteria count (total Lactic acid bacteria = 6.9 cfu/g), and simultaneous total yeast and mold count at 0.1 cfu/g. Table 1 presents the chemical analysis of the tested silage.

At the beginning of the trial, pigs across all three groups had comparable body weights (average initial mean body weight: 59.47 ± 0.85 kg). Each group comprised six animals (three gilts and three barrows). From a husbandry perspective, pigs were housed in semi-slatted concrete–floor pens within a controlled-environment facility. During the finishing period, the temperature was maintained between 18–23 °C, relative humidity between 50–70%, and ventilation rates were adjusted to avoid draughts, while maintaining air speed below 0.3–0.5 m/s. Stocking density was 1.1 m^2^/pig, in accordance with EU legislation (Council Directive 2008/120/EC). Standard farm management procedures were followed, including vaccination. Feed and water were provided ad libitum. Pigs in the control group (Silage-0%) were offered a commercial diet formulated according to National Research Council guidelines [26] and the Premier Nutrition database [27], whereas the experimental diets included the tested silage at a 5% (Silage-5%) or 10% (Silage-10%) inclusion rate. The diets were balanced to be isonitrogenous and isocaloric. In Table 2, the composition of diets and the chemical analysis of the final feeds are presented. The trial lasted 60 days (from 120 to 180 days of age). Pigs were individually weighed at day 1 and 60 days later, when the experiment ended, using a Mini–L 3510 animal scale (Zygisis, Chalkidiki, Greece). Daily records were kept of feed intake, water consumption, and mortality rates. Zootechnical performance indices were calculated, including average weight gain (AG, kg/period), average feed intake (AFI, kg feed/period), and feed conversion ratio (FCR, kg feed intake/kg live weight gain). On day 60, blood samples were collected from six pigs per group before slaughter. Animals were humanely slaughtered in an abattoir near the experimental premises. Carcass samples of shoulder (triceps brachii) and pancetta (external abdominal muscle) were collected, along with intestinal samples (ileum and caecum), which were obtained aseptically during evisceration.

### 2.2. Phenolic Content and Thiobarbituric Acid Reactive Substances (TBARs) Determination in Animal Feed

The total phenolic content of the diets was determined using the Folin–Ciocalteu assay, following the procedure of Vasilopoulos et al. [28]. Lipid oxidation was evaluated using a thiobarbituric acid–reactive substances (TBARS) assay, modified from the method of Botsoglou et al. [29]. In brief, 1.0 g of feed was homogenized with 8 mL of 5% (*w/v*) trichloroacetic acid (TCA) and 5 mL of 0.8% (*w/v*) butylated hydroxytoluene (BHT) prepared in hexane. The homogenate was centrifuged at 3000× *g* for 5 min, and 1.5 mL of the resulting aqueous phase was collected. This fraction was mixed with 2.5 mL of 0.8% (*w/v*) thiobarbituric acid (TBA) and incubated in a water bath at 70 °C for 30 min. For the estimation of the values for TBARs, a standard curve was prepared using standard solutions of 1,1,3,3-tetraethoxypropane, a precursor of malondialdehyde. The absorbance was recorded at 532 nm, with lipid oxidation reported as TBARS in mg of malondialdehyde (MDA) per kg of feed.

### 2.3. Isolation, Enumeration and Identification of Bacterial Populations

Fresh ileal and caecal digesta were obtained immediately post-slaughter from six pigs per dietary treatment. For primary processing, 1 g of intestinal content was homogenized in 9 mL of sterile 0.1% (*w/v*) peptone water. Quantitative bacteriology followed the Miles–Misra surface-drop technique: twelve-step, tenfold serial dilutions (10^−1^–10^−12^) were prepared in 96-well microplates, and 10 µL aliquots from each dilution were spotted onto the appropriate media. MacConkey agar and Kanamycin Aesculin Azide (KAA) agar (Merck, Darmstadt, Germany) were used to recover *Enterobacteriaceae* and *Enterococci*, respectively; plates were incubated aerobically at 37 °C for 24–48 h. Lactic acid bacteria were enumerated on De Man, Rogosa and Sharpe (MRS) agar (Oxoid, Basingstoke, UK) and on M17 agar (Lab M Limited, Lancashire, UK), with incubation at 37 °C for 48 h under anaerobiosis. *Bifidobacteriaceae* were quantified on Transgalactooligosaccharide Propionate (TOS) agar (Merck, Darmstadt, Germany) supplemented with glacial acetic acid (1%, *v/v*) and mupirocin (100 µL/mL), incubated anaerobically at 37 °C for 72 h. Total aerobic and anaerobic counts were estimated on plate count agar (Oxoid) after incubation at 30 °C for 48 h (aerobic) and at 37 °C for 48–72 h (anaerobic), respectively. Characteristic colonies from countable spots were recorded using a colony counter, expressed as log_10_ CFU per g of wet digesta, then described and subcultured. Taxonomic assignment at the family level was performed with the VITEK^®^ 2 compact system (bioMérieux, Marcy l’Etoile, France), which provides reliable identification across a broad range of Gram-positive and Gram-negative bacteria [30]. For *Enterobacteriaceae*, *Enterococcaceae*, *Lactobacillaceae,* and *Bifidobacteriaceae*, the VITEK^®^ 2 ID-GN, ID-GP, CBC/ANC, and ANC ID cards (bioMérieux) were used, respectively.

### 2.4. Hematological and Biochemical Analysis of the Blood

On day 60 (final sampling, pre-slaughter), blood was obtained from six pigs per dietary group following a 4 h feed withdrawal. Approximately 4 mL was collected by jugular venipuncture into EDTA-treated vacutainer tubes for hematology. Hematological indices—hemoglobin concentration, erythrocyte count, hematocrit (HCT), total leukocytes, and lymphocytes—were quantified using the MS4 (Melet Schloesing Lab, Osny, France) automated analyzer. Serum biochemical variables—albumin (ALB), alanine aminotransferase (ALT), aspartate aminotransferase (AST), cholesterol (CHOL), creatine kinase (CK), glucose (GLU), total bilirubin (TBIL), and triglycerides (TRIG)—were measured with a VetTest 8008 analyzer (IDEXX Laboratories, Westbrook, ME, USA).

### 2.5. Meat Chemical and Color Analysis

Triceps brachii and external abdominal oblique muscle samples were excised and immediately frozen at −20 °C for subsequent analyses. Portions of 200 g were comminuted in a heavy-duty meat grinder (Bosch, Gerlingen, Germany). Near-infrared spectroscopy (FoodScan™ Lab, FOSS, Hillerød, Denmark) was used to quantify moisture, crude protein, fat, collagen, and ash, following AOAC 2007.04 for meat and meat products [31].

Muscle pH (triceps brachii and external abdominal oblique meat cuts) was measured with a portable pH meter designed for solid matrices (HI981036; Hanna Instruments, Woonsocket, RI, USA). For each dietary group, six pork samples were assessed by inserting the stainless-steel probe into the geometric center of the tissue; group means were then calculated.

Color measurements of the triceps brachii and external abdominal oblique muscles were conducted using a Hunter L *, a *, b * color system with a CAM-System 500 chromameter (Lovibond, Amesbury, UK) [22]. Meat samples, cut to a thickness of 1.2 cm, were placed on polystyrene trays and allowed to bloom for 30 min at 4 °C under aerobic conditions prior to analysis. Color parameters were recorded in the CIE Lab * space, where L * corresponds to lightness, a * to red–green intensity, and b * to yellow–blue intensity.

### 2.6. Oxidative Stability Analysis of the Meat

A modified Folin–Ciocalteu assay was used to quantify total polyphenols in fresh pork tissues (3rd day after slaughter). A 0.2 g/L gallic acid stock (Merck, Germany) was prepared in 100 mL distilled water and serially diluted to generate standards of 0.005, 0.01, 0.05, 0.1, 0.25, 0.5, and 1.0 g/L. For each calibrant, 0.2 mL was dispensed into a 50 mL polypropylene tube and combined with 10.8 mL distilled water, 8.0 mL sodium carbonate solution (75 g Na_2_CO_3_ per L; Penta Chemicals, Prague, Czech Republic), and 1.0 mL Folin–Ciocalteu reagent (PanReac AppliChem, Darmstadt, Germany). A reagent blank contained 0.2 mL distilled water in place of standard. After vortex mixing, tubes were incubated for 1 h at room temperature in the dark. Absorbance was recorded at 750 nm on a DR 5000 UV–Vis spectrophotometer (Hach Lange, Düsseldorf, Germany), using the blank for instrument zeroing. The calibration curve (absorbance vs. gallic acid concentration) was linear with R^2^ = 0.9989 (constructed in Microsoft Excel). Meat extracts were prepared by homogenizing 5 g of shoulder, belly, or ham with 10 mL distilled water, followed by filtration through paper. Aliquots (0.2 mL) of filtrate were reacted identically to the standards, with a matrix blank (0.2 mL water) used for spectrophotometer calibration. Results were expressed as gallic acid equivalents.

Lipid oxidation was quantified using a thiobarbituric acid reactive substances (TBARS) assay, as modified based on Dias et al. [32], on the 3rd day after slaughter. For this procedure, 5 g samples of triceps brachii or external abdominal oblique muscle were homogenized with 25 mL of trichloroacetic acid (TCA), transferred to glass containers, and allowed to stand for 20 min. The homogenate was then filtered, and 5 mL of the resulting filtrate was mixed with 5 mL of 2-thiobarbituric acid (TBA) solution in glass tubes. A reagent blank was prepared by replacing the filtrate with 5 mL of TCA. After vortexing, tubes were incubated in a water bath at 60 °C for 15 min and cooled to room temperature, and the absorbance was read at 532 nm on a UV–Vis spectrophotometer using the blank for baseline correction. For the estimation of TBARs values, a standard curve was prepared using standard solutions of 1,1,3,3-tetraethoxypropane.

### 2.7. Microbiological Analysis of Meat Cuts

Following 48 h of refrigerated storage at 4 °C (to accommodate transport and handling), microbiological analyses were conducted on meat from six pigs per treatment. For each sample, 10 g of shoulder or belly meat was homogenized in a BagMixer 400 (Interscience, Saint-Nom-la-Bretèche, France) with 90 mL of sterile Maximum Recovery Diluent (MRD; Oxoid, Basingstoke, UK) to obtain a 10^−1^ suspension. Tenfold serial dilutions were then prepared in 9 mL MRD, and appropriate dilutions (1.0 or 0.1 mL) were plated for enumeration as follows: *Escherichia coli* on Tryptone Bile X-Glucuronide (TBX) agar at 37 °C for 24 h (aerobic); sulfite-reducing clostridia on Perfringens Agar Base at 37 °C for 48 h under anaerobiosis generated with Anaerocult A (all Oxoid); *Staphylococcus aureus* and other *Staphylococcus* spp. on Baird–Parker agar supplemented with egg-yolk tellurite (50 mL L^−1^) at 37 °C for 48 h (aerobic); total mesophilic counts on Plate Count Agar at 30 °C for 48 h (aerobic); and *Campylobacter jejuni* on Campy Blood-Free Selective Medium (CCDA; Acumedia–Lab M, Lansing, MI, USA) with selective supplement under microaerophilic conditions (10% CO_2_) at 37 °C for 72 h. Detection of *Salmonella* spp. and *Listeria monocytogenes* was performed on 25 g portions in accordance with ISO 6579:2002 [33] and ISO 4833:2001 [34], respectively. All plates were incubated in thermostatic cabinets (BD 115; Binder, Germany).

### 2.8. Fatty Acid Analysis of the Meat

Fatty acids in triceps brachii and external abdominal oblique muscles were analyzed after in situ transesterification according to the method described by O’Fallon et al. [35]. Fatty acid methyl esters (FAME) were separated and quantified following the procedure of Giannenas et al. [36] on a TraceGC gas chromatograph (Model K07332; ThermoFinnigan/Thermoquest, Milan, Italy) equipped with a flame ionization detector. Retention times and elution order were assigned using the Supelco “37 Component FAME Mix” reference standard (Sigma-Aldrich, Darmstadt, Germany). Individual fatty acids were expressed as a percentage of total identified FAME (peak area of the analyte divided by the sum of peak areas for all identified fatty acids). From these data, the PUFA/SFA ratio, the n-6/n-3 PUFA ratio, and the hypocholesterolemic-to-hypercholesterolemic fatty acid index (h/H) were calculated, where h/H = (C18:1n-9 + ΣPUFA)/(C12:0 + C14:0 + C16:0). The h/H index was used as an indicator of the predicted cholesterolemic impact of the lipid fraction [37].

### 2.9. Statistical Analysis

A randomized complete block design (RCB) was employed, considering each ear-tagged pig to be an experimental unit. Microbiological data were log_10_-transformed prior to statistical analysis. Homogeneity of variances was assessed using Levene’s test. Depending on data distribution, either one-way ANOVA (parametric) or the Kruskal–Wallis test (non-parametric) was applied in SPSS v20 [38]. When ANOVA revealed a significant treatment effect (*p* ≤ 0.05), mean separation among the three dietary treatments was conducted using Tukey’s HSD test. Statistical significance was set at α = 0.05 for all analyses.

## 3. Results

### 3.1. Total Phenolic Content and Lipid Oxidation of the Control and Experimental Diets

Total phenolic content value in the control group (Silage-0%) feed was significantly lower (*p* = 0.027) than in both treatment groups (Silage-5% and Silage-10%). A similar positive pattern was noticed for Malondialdehyde (MDA), which was used as an indicator in evaluating the degree of feed lipid oxidation. MDA levels significantly (*p* = 0.001) deteriorated in all the silage-enriched diets compared to the control Silage-0% group (Table 3).

### 3.2. Performance and Carcass Parameters

The effects of silage supplement on pig performance and carcass parameters are presented in Table 4. The final live weight and total weight gain of the finishing pigs did not differ (*p* = 0.281 and *p* = 0.659, respectively) between the three treatments. Feed intake (FI) and feed conversion efficiency (FCR) remained within the normal production ranges reported for the intensive pig production unit. Concerning the carcass parameters, carcass weight was similar for all groups, though arithmetically better for the Silage-10% animals, but the dressing percentage was significantly (*p* = 0.019) increased in Silage-5% and Silage-10% groups.

### 3.3. Intestinal Microflora

The dietary use of the tested silage affected intestinal microflora populations (Table 5). In the ileum digesta, it was noted that *Enterobacteriaceae* were significantly reduced (*p* = 0.001) in Silage-10% and Enterococci were reduced in both Silage-5% and Silage-10%. Furthermore, Lactobacilli were significantly increased (*p* = 0.001) in treatments Silage-5% and Silage-10% compared to the control. Total anaerobes were at the lowest level (*p* = 0.038) in the caecum digesta, in the experimental diet (Silage-5% treatments), compared to Silage-10% treatment. Enterococci levels were significantly (*p* = 0.001) lowered and Lactobacilli tended to increase, but only numerically, in both of the silage-enriched diets. *Bifidobacterium* was not affected (*p* = 0.632) by the treatments in any of the intestinal samples.

### 3.4. Hematological and Biochemical Parameters

Concerning hematological values, the pigs in treatment groups Silage-5% and Silage-10% tended (*p* = 0.060) to have higher monocyte levels compared to the control Silage-0% group. Regarding the biochemical parameters, blood alanine aminotransferase (ALT) was diminished in both silage-enriched treatments, but this reduction was only significant in group Silage-10% (*p* = 0.030) compared to control. Blood hematological and biochemical parameters generally did not differ (*p* > 0.05) between the three treatments. Table 6 presents the effects of the silage on the pigs’ blood hematological and biochemical parameters.

### 3.5. Meat Analysis

As shown in Table 7, no differences were noted in the chemical composition of the triceps brachii muscle samples for all silage treatments. Regarding the external abdominal oblique samples, moisture content was significantly decreased (*p* = 0.020) in treatment Silage-10%, but only when compared to Silage-5% group, where a slight elevation was noticed. All other parameters examined (fat, protein, collagen, and ash) were not affected (*p* = 0.930, *p* = 0.450, *p* = 0.740 and *p* = 0.150, respectively) by the treatments. The pH values of all samples for both shoulder and belly meat did not differ between the treatments (*p* = 0.590).

As shown in Table 8, colorimetric analysis revealed a significant increase (*p* = 0.001) in redness (a * value) of the triceps brachii muscle in both silage-supplemented groups (5% and 10%), while simultaneously, there was a significant (*p* = 0.049) L * value reduction in the experimental groups. No statistical differences (*p* > 0.05) were noticed in the external abdominal oblique meat samples.

The results of the meat microbiological analysis are presented in Table 9. In the triceps brachii, *Campylobacter jejuni* was lower (*p* = 0.039) in the Silage-10% treatment compared to the control. Total Mesophilic count, *Staphylococcus aureus*, *Staphylococcus* spp., *Escherichia coli*, and Sulfite-reducing *Clostridium* did not differ (*p* > 0.05) between the treatments in triceps brachii and external abdominal oblique samples. *E. coli* counts in external abdominal oblique meat ranged between 1.04 and 1.46 log CFU/g, corresponding to approximately 10–30 CFU/g. All meat cuts tested negative for *Salmonella* spp. and *Listeria monocytogenes* (per 25 g of sample).

Table 10 presents data on the total phenolic content and oxidative stability of the meat samples. A significantly higher phenolic content (*p* = 0.013) was observed in triceps brachii meat from the Silage-10% group compared to the control group. Total phenols were not significantly affected by treatment in the external abdominal oblique meat samples. Thiobarbituric acid reactive substances (TBARS) did not differ in any of the meat cuts (*p* = 0.201 and *p* = 0.171 for triceps brachii and external abdominal oblique, respectively) and showed very low values (almost an absence) of lipid oxidation, since all samples were fresh and not stored for a prolonged period of time (3rd day after slaughter).

Fatty acid profiles of the triceps brachii cuts are presented in Table 11. Dietary supplementation with the tested silage significantly (*p* < 0.05) altered several fatty acid indices in the 5% and 10% groups compared with the 0% control group. Specifically, total saturated fatty acids (Σ SFA) were lowest (*p* = 0.049) in the Silage-5% group, intermediate in the Control-0% group, and highest in the Silage-10% group. Total monounsaturated fatty acids (Σ MUFA) were highest (*p* = 0.021) in the Silage-5% group, intermediate in the Silage-10% group, and lowest in the Control-0% group. Total polyunsaturated fatty acids (Σ PUFA) did not differ between the Silage-5% and Control-0% treatments but were reduced (*p* = 0.029) in the 10% group.

Fatty acid results for the external abdominal oblique cuts are shown in Table 12. In this case, dietary silage supplementation affected only a limited number of fatty acids and indices. In particular, total MUFA were reduced (*p* = 0.043) in the Silage-10% group, and the n-6/n-3 fatty acid ratio was significantly decreased (*p* = 0.005) in the same treatment.

## 4. Discussion

Greece generates substantial quantities of agro-industrial by-products due to its strong and regionally concentrated production of wine, olive oil, and dairy products—three pillars of the Mediterranean agri-food sector. The country ranks among the leading EU producers of table olives, olive oil, and feta cheese, and maintains a long tradition of small- to medium-scale wineries. As a result, large seasonal volumes of grape pomace, olive-mill wastewater, and cheese whey are produced annually. Although often treated as waste, these streams contain valuable bioactive components such as polyphenols, organic acids, fibers, residual sugars, and antioxidant compounds. Their biological significance lies in their potential to enhance gut health, modulate oxidative balance, and improve meat quality when appropriately processed and included in animal feed [1,10,11,24]. Consequently, valorizing these abundant by-products through controlled fermentation or silage production offers both an environmental management strategy and an opportunity for functional feed innovation in Mediterranean livestock systems. It is estimated that the annual production of fresh grape pomace (GP), olive-mill wastewater (OMWW), and cheese whey (CW) in Greece is about 200,000 tons, 0.8–1.2 × 10^6^ tons and 1.0–1.3 × 10^6^ tons, respectively [39]. Thus, it becomes evident that there are considerable amounts of agri-food waste that are potential pollutants and could be recycled and reused in modern livestock production. This trial evaluated for the first time a novel silage manufactured from three Greek agro-industrial by-products—olive-mill wastewater solids, grape pomace solids, and de-proteinized feta whey—fed at 0%, 5%, and 10% inclusion levels in finishing-pig diets. The concept aligns with recent circular-bioeconomy approaches that have formulated silages or feeds from exactly these streams and tested them in poultry and swine, demonstrating technical feasibility and safety while aiming to reduce dependence on conventional grains and soybean meal [15,19,20,21,22]. Within this framework, our data show that (i) productive performance, final weight, feed conversion efficiency and carcass weight were preserved across treatments, while dressing percentage increased at 5 and 10% inclusion rates; (ii) ileal eubiosis indicators improved (↓ Enterobacteriaceae, ↓ Enterococci; ↑ Lactobacilli), with more modest changes observed in the caecum; (iii) serum biochemistry remained largely within reference limits with a modest reduction in ALT at 10%; and (iv) meat proximate composition and pH were unaffected, triceps brachii became redder (↑ a *) and slightly darker (↓ L *), Campylobacter jejuni counts decreased in shoulder cuts, and (v) the fatty acid contents remained generally unchangeable and the n-6/n-3 ratio in muscle fat showed a slightly improvement in the Silage-10% group.

All of these parameters evaluate the usage of agricultural by-products in monogastric animals, specifically pigs, without adverse effects being recorded in any zootechnical, health, or meat quality parameters. This allows for significant improvements in the silage or by-products characteristics in order to achieve beneficial results in the fatty acid profile and the antioxidant capacity of meat, as well as in the microbial populations in the gut of fattening pigs, with the addition of higher percentage of agro-industrial by-products.

### 4.1. Feed Antioxidant Metrics and Lipid Peroxidation

Feed MDA was lower in both silage-enriched diets (5% and 10%) than in the control, with higher dietary total phenolic content also recorded in the former. This pattern is consistent with the in-matrix antioxidant activity of grape- and olive-derived polyphenols, which can quench lipid radicals and chelate pro-oxidant metals, thereby interrupting propagation steps of lipid peroxidation in the feed itself. Comparable outcomes—reduced oxidative indices and/or enhanced phenolic density of the diet—have been reported when sole winery or olive coproducts (grape pomace or olive leaves, or OMWW extracts) were incorporated into monogastric rations or used to fortify food/feed matrices. These interventions frequently lower MDA or peroxide values in the matrix and/or downstream animal products, or at minimum, maintain oxidative status despite higher unsaturation loads [15,40,41]. In pigs, phytogenic/phenolic preparations from olive mill wastewater have repeatedly reduced TBARS (and protein carbonyls) in vivo, supporting the notion that OMWW phenolics exert meaningful antioxidant effects within swine systems [42]. Physiologically, a less oxidized diet can reduce the oxidative challenge entering the gastrointestinal tract, which may help explain the favorable ileal shifts we observed (↓ *Enterobacteriaceae*/Enterococci; ↑ Lactobacilli), showing absence of inflammatory indices; this was also observed in the biochemical and hematological results, in addition to the absence of pro-oxidative signals in meat despite modest fatty-acid changes. While much of the oxidized-feed literature is poultry-centric, it consistently shows that controlling feed oxidation improves redox balance and product quality—principles that plausibly extend to pigs as monogastrics [43]. In our case, the combination of lower feed TBARS, unchanged meat TBARS, and redder shoulder color (↑ a *) suggests that the silage’s phenolic fraction chiefly protected the diet and may have contributed to a stable post-absorptive oxidative environment, in line with prior reports on grape/olive polyphenols in monogastric nutrition [40,41,42,44]. In finishing pigs, substituting part of the fat with olive-pomace acid oil elevated product oxidation at certain inclusion levels compared with refined blends, underscoring that the source and handling of ingredients modulate oxidative outcomes [45]. At the same time, polyphenol supplementation in pigs yields heterogeneous antioxidant results—some studies show reduced TBARS in plasma or meat, others show no change—depending on extract type, dose, basal diet, and sampling matrix [46,47]. For instance, Gessner et al. reported no differences in plasma or liver TBARS in pigs fed grape seed/grape marc meal extract, despite anti-inflammatory intestinal effects [48]. Conversely, classical α-tocopherol trials consistently lowered pork TBARS during storage, illustrating that not all antioxidants behave identically in vivo or in feed [49]. In our study, the lack of treatment differences in meat TBARS—despite lower feed MDA—suggests that (a) dietary phenolics and/or improved gut ecology may have protected muscle lipids post-absorption, (b) the freezing/holding regime truncated oxidative divergence, or (c) the sampling window was too narrow to detect downstream effects. Similar null effects in pork TBARS with botanical phenolics have been reported when storage conditions were not strongly pro-oxidant [50,51,52].

### 4.2. Performance and Carcass Traits

Final live weight, ADG, and FCR did not differ among A, B, and C and were within the farm’s expected range, matching numerous finishing-pig studies where grape or olive by-products at modest inclusion preserved growth [3,4,5,6]. For example, replacing wheat bran with grape pomace improved water-holding traits but not growth in finishing pigs [53]. Additionally, dehydrated grape pomace at 5–10% levels maintained performance while altering some carcass and fat traits in a local genotype [51]. For olive coproducts, both neutral and beneficial effects on growth and carcass have been observed depending on inclusion level and processing (e.g., partially de-stoned or ensiled olive cake) [54,55,56,57,58]. We observed a higher dressing percentage at 5–10% silage. While not universal, increased dressing has been reported with phenolic-rich additives (e.g., grape seed proanthocyanidins or ferulic acid mixtures) in finishing pigs, potentially via lean-to-fat repartitioning and water losses at slaughter [59,60]. Conversely, some olive-cake programs reported unchanged dressing or minimal at the highest inclusions, indicating that matrix, dose, and genotype interact [54,56]. Within that landscape, our response—an improved dressing percentage without weight penalty—fits within the “no-harm to growth, occasional carcass benefit” envelope reported for moderate inclusions of winery/olive by-products [55,56].

### 4.3. Intestinal Microbiology

The ileal shift toward eubiosis (↓ *Enterobacteriaceae* and Enterococci; ↑ Lactobacilli) in B and C is consistent with the prebiotic and antimicrobial profile of plant polyphenols and fermentable fiber [61]. Grape pomace in pigs increased *Lactobacillus*/*Bifidobacterium* and modulated performance and antioxidant status, and broader reviews conclude that grape phenolics in pigs enhance antioxidant capacity, immune tone, and gut biodiversity [62]. Mechanistically, polyphenols can reduce pathobiont abundance through direct bacteriostatic or bactericidal action and by shifting substrates toward commensal fermenters; several pig studies and reviews support these pathways [61,62]. Our silage also contributed whey solids to the diet; independent studies show improved growth and beneficial fecal microbiota when liquid whey is included in pig diets [63,64]. Beyond single ingredients, finishing pigs fed non-conventional mulberry silages exhibited improved meat quality via gut-microbiota modulation and barrier integrity, supporting a “silage-to-gut-to-meat” axis [65]. Finally, closely related work with exactly this innovative silage in pigs demonstrated microbiota compositional shifts without performance penalties, reinforcing our enteric findings [21].

### 4.4. Serum Biochemistry

The modest reduction in ALT in the Silage-10% group is directionally compatible with hepatoprotective effects of certain plant polyphenols under oxidative or inflammatory challenge in piglets (e.g., holly leaf Ilex latifolia polyphenols attenuating LPS/diquat-induced liver injury with lower ALT/AST/GGT). However, in non-challenged settings, olive or grape polyphenols often leave transaminases unchanged [66]. Thus, our small ALT decrease should be interpreted as biologically modest but not inconsistent with a mild improvement in hepatic redox/immune milieu.

### 4.5. Meat Composition, pH, and Color

The proximate composition and pH of triceps brachii and external abdominal muscles were unaffected, as commonly observed with moderate botanical inclusions. Triceps brachii became redder (↑ a *) and slightly darker (↓ L *) in B and C. Although color can be largely affected by parameters like pH, water content and water holding capacity, and myglobin concentration and redo/oxidative state, these characteristics was not altered after the applied treatments. It seems that the increased redness resulted from the addition of antioxidant compounds in the bioactive feed supplement (silage). Antioxidants can stabilize oxymyoglobin and mitigate metmyoglobin formation, thereby sustaining redness. Phenolics may also interact with muscle iron chemistry and endogenous enzymes, contributing to color stability, while darker appearance (lower L *) can arise from subtle differences in water distribution or myoglobin state. Reports with grape products or antioxidant mixtures in pigs similarly describe improved early post-mortem redness and/or water holding without large pH shifts [53,59]. The observed decrease in moisture percentage in the pork belly meat cuts can be attributed to the elongation of fatty acids towards monosaturated and unsaturated, with the simultaneous increase in fat content functioning to advertise possible flavor and texture shifts; it is possible to differentiate these cuts according to consumers’ selection criteria.

### 4.6. Meat Microbiology

The lower *Campylobacter jejuni* counts in the shoulder in the 10% group dovetail with extensive in vitro evidence that phenolic extracts—including grape seed and sorghum phenolics—and even olive-mill wastewater polyphenols inhibit *Campylobacter* growth, adhesion, and biofilm formation [67]. While most intervention data are post-harvest or in poultry systems, the directionality supports a plausible diet-to-muscle reduction mediated either by (i) lower intestinal/lymphatic carriage at slaughter or (ii) carry-over of phenolics to the muscle microenvironment where they can exert antimicrobial effects. The absence of *Salmonella* and *L. monocytogenes* across treatments is foremost an indicator of sound hygiene; polyphenols can suppress these pathogens in meat systems, but our experimental design cannot attribute this absence to the diet. The sporadic *E. coli* in the belly (but not the shoulder) is consistent with anatomical contamination risk rather than a treatment effect.

### 4.7. Phenolic Content in Meat and Oxidative Stability

The detected higher total phenolics in triceps brachii at 10% silage are consistent with reports that dietary plant phenolics (especially from grape) can increase meat total phenolics and antioxidant capacity in monogastrics, though the magnitude varies with extractability and conjugation [66]. Meat TBARS did not differ among groups, despite higher feed MDA; this outcome is congruent with several pig studies in which grape-derived phenolics either reduced systemic lipid peroxidation or left TBARS unchanged at slaughter, depending on dose and storage protocol [50,51]. Together with improved redness, unchanged TBARS points to adequate oxidative protection of muscle lipids at the tested inclusion levels and under our storage conditions.

### 4.8. Fatty-Acid Profile

It is well-established that the fatty acid profiles of meat lipids in pigs and poultry closely reflect the composition of fats in their diets [68,69]. Dietary enrichment with polyunsaturated fatty acids is often linked to elevated levels of these acids in the muscle and adipose tissues, both through direct incorporation and modification of unsaturated fatty acids synthesis, by modulating lipid metabolism through the suppression of key lipogenic enzymes involved in de novo fatty-acid synthesis [70]. Thus, the formulation of the silage containing a high proportion of OMWW [19] may explain the improved (*p* = 0.005) n-6/n-3 ratio observed in the Silage-10% group (17.54 → 15.09), reflecting a modest yet favorable shift in muscle lipid quality in the external abdominal oblique cuts, despite the concurrent reduction in total MUFA (*p* = 0.049). Reviews and trials document that grape by-products can beneficially influence pork FA profile (including modest n-3 increases) and that certain olive-cake programs shift MUFA upward and, in some cases, lower dietary n-6/n-3 [48,51,53]. Moreover, controlled manipulations of the dietary n-6/n-3 ratio in pigs produce corresponding shifts in tissue FA and can improve meat-quality indices without compromising oxidative stability when properly balanced. Another study reported that inclusion of grape pomace solids at a 5% rate in finishing-pig diets did not affect the SFA, MUFA, PUFA, n-6 or n-3 PUFA percentages in the meat [51,71,72]. Although the innovative silage used was not a n-3 supplement per se, the aggregate of olive- and grape-derived lipids plus secondary metabolic effects may explain the modest reduction observed. If a larger decrease is targeted, co-inclusion of known n-3 sources (e.g., linseed oil) with grape/olive by-products could be effective and widely reported [73]. Finally, ensiled olive cake at high inclusion has been shown to improve pork nutritional indices while valorizing a key Mediterranean by-product [53].

Overall, the data support that a 5–10% inclusion of this Greek mixed silage can (i) maintain finishing performance; (ii) modestly improve carcass dressing; (iii) promote ileal eubiosis with lower *Enterobacteriaceae*/Enterococci and higher Lactobacilli; (iv) produce redder triceps brachii meat without compromising pH, proximate composition, or TBARS; (v) reduce *C. jejuni* recovery in triceps brachii meat; and (vi) slightly improve the n-6/n-3 ratio in muscle lipids. These outcomes are consistent with prior work on weaned pigs and microbiomes using the same silage concept and with broader evidence on grape/olive phenolics in pigs [18,19]. The study scope and boundary conditions included a modest sample size, assessment within a single finishing cycle and genotype, and potential matrix effects specific to the silage (e.g., phenolic spectrum, degree of fatty-acid unsaturation, and mineral load) that may influence feed oxidation indices without necessarily manifesting in meat outcomes. The results are also expected to depend on ingredient composition, inclusion level, and processing conditions; any scale-up should additionally consider regulatory context, cost, and palatability. Future work could incorporate extended storage simulations (e.g., refrigerated retail display) and targeted lipidomics to characterize oxidative resilience, while metagenomic and metabolomic profiling would clarify microbe–phenolic interactions along the gut–muscle axis. Finally, evaluating defined n-3 co-supplementation strategies to enhance the favorable shift in the n-6/n-3 ratio—concurrent with sensory assessment—would help optimize nutritional and product-quality endpoints.

## 5. Conclusions

In contemporary animal production, developing alternative feed sources is increasingly important for reducing reliance on conventional ingredients, lowering costs, increasing sustainability, and recycling agro-industrial residues. The present study evaluated, for the first time in finishing pigs, a silage produced from olive, winery, and cheese by-products incorporated at 0%, 5%, and 10% of the diet. The inclusion of this silage did not negatively affect growth performance, carcass traits, hematological indices, or general meat quality. Instead, beneficial effects were observed on intestinal microbiota composition, with increased Lactobacilli and decreased *Enterobacteriaceae* and Enterococci, together with elevated muscle phenolic content and an improved n-6/n-3 fatty acid ratio at higher inclusion levels. Although lipid oxidation (TBARS) was not significantly altered, meat color and some microbial parameters, such as *Campylobacter jejuni* prevalence, were favorably influenced. Overall, these findings indicate that the investigated silage supports pig health and meat functionality while simultaneously valorizing significant Greek agro-industrial by-products, thereby contributing to more circular and environmentally sustainable livestock production.

## Figures and Tables

**Table 1:** Chemical composition of the evaluated silage.

Silage Chemical Analysis (As-Fed Basis)	
Moisture (%)	42.89
Dry matter (%)	57.11
Ash (%)	1.15
Crude fat (%)	3.21
Crude fiber (%)	2.63
Crude protein (%)	5.51
Total Ca (%)	0.05
Total P (%)	0.18
Mn (mg/kg)	16.95
Fe (mg/kg)	82.48
Cu (mg/kg)	3.21
Zn (mg/kg)	30.43

**Table 2:** Composition and calculated proximate analysis of experimental diets.

Diets			
**Ingredients (%)**	**Silage-0%**	**Silage-5%**	**Silage-10%**
Wheat	45.00	37.49	29.98
Silage	0.00	5.00	10.00
Soybean meal (47% CP)	14.00	15.57	17.14
Barley	27.60	27.60	27.60
Wheat middlings	9.50	9.50	9.50
Soybean oil	1.15	2.09	3.03
Commercial premix ^1^	1.00	1.00	1.00
Amino-acid Premix ^2^	0.25	0.25	0.25
Salt	0.50	0.50	0.50
Limestone	1.00	1.00	1.00
**Total**	**100.00**	**100.00**	**100.00**
**Calculated chemical analysis**			
Digestible energy, MJ/kg	13.339	13.339	13.339
Crude protein, %	16.553	16.750	16.946
Dry matter, %	87.684	86.331	84.977
Ash, %	4.557	4.571	4.584
Crude fat, %	2.425	3.377	4.329
Crude fiber, %	3.878	3.861	3.844
ADF, %	4.620	4.588	4.555
NDF, %	13.653	13.448	13.243
Ca, %	0.568	0.571	0.574
Total P, %	0.385	0.386	0.386
Lysine, %	1.072	1.102	1.131
Methionine + Cystine, %	0.615	0.615	0.615
Threonine, %	0.747	0.762	0.776
Tryptophan, %	0.232	0.234	0.235

^1^ Provided per kg complete diet: 6500 IU retinyl acetate; 1200 IU cholecalciferol; 12.5 mcg 25-hydroxycholecalciferol; 60 mg alpha-tocopherol acetate; 2 mg menadione nicotinamide bisulphite; 2 mg thiamine mononitrate; 7 mg riboflavin; 25 mg pantothenic acid; 3 mg pyridoxine hydrochloride; 25 mcg cyanocobalamin; 25 mg nicotinic acid; 1 mg folic acid; 0.15 mg biotin; 300 mg choline chloride; 108 mg Fe from ferrous sulphate monohydrate; 25 mg Cu from copper sulphate; 48 mg Mn from manganese oxide; 84 mg Zn from zinc oxide; 1.2 mg I from calcium iodate; 0.24 mg Se from sodium selenite; 700 mg methionine; 100 mg L-tryptophan; 2730 L-Lysine mg HCl; 1182.02 mg L-threonine; 1500 FYT 6-fytase; 200 FXU endo-1,4-β-xylanase. ^2^ Provided per kg complete diet in group A: 871.88 mg L-lysine HCl; 824.74 mg L-threonine; 98.87 mg L-tryptophan; and 44 mg DL-methionine.

**Table 3:** **Total Phenolic** **(TP) content and MDA in pig diets.**

	Diets			SEM *	*p*-Value
	Silage-0%	Silage-5%	Silage-10%		
**TP (mg GAE/L Feed extract)**	139.41 ^a^	153.87 ^b^	172.44 ^c^	5.130	0.027
**mgMDA/Kg Feed**	0.091 ^c^	0.066 ^b^	0.046 ^a^	0.006	<0.001

^a,b,c^ Values with no common superscript differ significantly (*p* ≤ 0.05); n = 18 (6 pigs per diet). * Standard error of the mean.

**Table 4:** Effect of silage supplementation on fattening pigs’ performance and carcass indices.

	Diets			SEM	*p*-Value
**Performance Parameters**	**Silage-0%**	**Silage-5%**	**Silage-10%**		
Initial bodyweight (kg)	57.75	59.48	61.18	0.850	0.272
Final bodyweight (kg)	122.08	123.6	124.03	1.510	0.281
Weight gain (kg)	64.33	64.11	66.76	1.250	0.659
FI/per pig * (kg)	187.72	189.15	191.15	NA	NA
FCR * (kg feed/kg weight gain)	2.92	2.95	2.86	NA	NA
**Carcass parameters**					
Carcass weight (kg)	72.08	73.47	73.94	0.876	0.138
Dressing percentage (%)	59.04 ^a^	59.44 ^b^	59.61 ^b^	0.002	0.019

* n = 18 (6 pigs per diet); ^a,b^ Values with no common superscript differ significantly (*p* ≤ 0.05); FI = Feed Intake; FCR = Feed Conversion Ratio; NA = Not applicable.

**Table 5:** Silage effects on fattening pigs’ intestinal microbial populations.

	Diets			SEM	*p*-Value
**Ileum Microbes (Log_10_ CFU/g)**	**Silage-0%**	**Silage-5%**	**Silage-10%**		
Total aerobes	9.10	8.77	8.16	0.168	0.060
Total anaerobes	8.80	8.88	8.55	0.111	0.484
*Enterobacteriaceae*	4.96 ^b^	5.14 ^b^	3.86 ^a^	0.171	0.001
Enterococci	5.96 ^c^	4.58 ^b^	3.46 ^a^	0.273	0.001
Lactobacilli	6.67 ^a^	8.16 ^b^	9.00 ^b^	0.269	0.001
*Bifidobacterium*	6.05	5.82	5.92	0.105	0.703
**Caecum microbes (Log_10_ CFU/g)**					
Total aerobes	9.19 ^ab^	8.96 ^a^	9.48 ^b^	0.086	0.038
Total anaerobes	9.25	8.90	9.02	0.095	0.344
*Enterobacteriaceae*	5.38	5.46	4.87	0.126	0.112
Enterococci	7.00 ^a^	3.75 ^b^	4.74 ^c^	0.341	0.001
Lactobacilli	8.75	9.64	9.46	0.178	0.090
*Bifidobacterium*	6.02	6.28	6.09	0.107	0.632

n = 18 (6 pigs per treatment); SEM: Standard error of the mean; ^a,b,c^ Values with no common superscript differ significantly (*p* ≤ 0.05).

**Table 6:** Silage effects on fattening pigs’ blood hematological and biochemical parameters.

	Diets			SEM	*p*-Value
**Hematological Parameters**	**Silage-0%**	**Silage-5%**	**Silage-10%**		
WBC (10^3^/μL)	20.71	18.89	20.31	0.720	0.590
Lymphocytes (%)	39.73	43.33	38.25	1.060	0.130
Monocytes (%)	7.28 ^x^	8.65 ^x^	9.18 ^y^	0.350	0.060
Granulocytes (%)	52.98	49.01	52.56	1.000	0.220
RBC (106/μL)	6.85	6.81	6.77	0.320	1.000
Hct (%)	38.4	36.15	35.13	1.740	0.760
Hb (g/dL)	11.66	12.2	11.88	0.380	0.870
THR (m/mm3)	237.66	224.33	240	14.660	0.910
**Blood biochemical parameters**					
ALP (u/L)	98.66	136.5	102.25	8.820	0.150
ALT (u/L)	70.33 ^b^	69.16 ^ab^	56.75 ^a^	2.460	0.030
AST (u/L)	32.66	43.83	42.16	3.790	0.450
CHOL (mg/dL)	87.5	98.83	88.41	2.530	0.170
GLU (mg/dL)	78.16	76.5	78.25	1.920	0.920
TRIG (mg/dL)	41.83	50	42.58	3.450	0.590
CK (u/L)	454.33	505.66	515.08	28.960	0.680

n = 18 (6 pigs per treatment); SEM: Standard error of the mean; ^a,b^ Values with no common superscript differ significantly (*p* ≤ 0.05); ^x,y^ Values with no common superscript tend to (0.05 < *p* ≤ 0.10); WBC: White blood cells; RBC: Red blood cells; Hct: Hematocrit; Hb: Hemoglobin; Albumin; ALP: Alkaline phosphatase; ALT: Alanine aminotransferase; AST: Aspartate aminotransferase; CHOL: Cholesterol; GLU: Glucose; TRIG: Triglycerides; CK: Creatine kinase.

**Table 7:** Silage effects on triceps brachii and external abdominal oblique meat chemical composition and pH.

	Diets				
Triceps Brachii Muscle Chemical Composition (%)	Silage-0%	Silage-5%	Silage-10%	SEM	*p*-Value
Fat	9.77	10.08	9.31	0.460	0.930
Moisture	69.74	69.69	69.6	0.350	0.990
Protein	19.97	19.92	20.24	0.320	0.450
Collagen	1.7	1.64	1.56	0.070	0.740
Ash	0.99	0.95	0.83	0.340	0.150
pH	5.52	5.56	5.54	0.220	0.820
**External abdominal oblique muscle Chemical Composition (%)**					
Fat	20.02	21.48	23.42	0.790	0.220
Moisture	60.54 ^ab^	60.91 ^a^	57.24 ^b^	0.640	0.020
Protein	18.93	17.4	18.44	0.410	0.30
Collagen	2.13	2.06	2.31	0.070	0.340
Ash	0.9	0.81	0.78	0.410	0.430
pH	5.55	5.51	5.53	0.020	0.590

n = 18 (6 pigs per treatment); SEM: Standard error of the mean; ^a,b^ Values with no common superscript differ significantly (*p* ≤ 0.05).

**Table 8:** Effect of silage addition on pigs’ triceps brachii and external abdominal oblique meat color.

	Diets			SEM	*p*-Value
**Triceps Brachii** **Meat Color**	**Silage-0%**	**Silage-5%**	**Silage-10%**		
L *	61.65 ^b^	55.3 ^a^	55.32 ^a^	1.259	0.049
a *	10.08 ^a^	16.78 ^b^	16.2 ^b^	0.947	0.001
b *	13.07	12.97	14.37	0.436	0.366
**External abdominal oblique meat color**					
L *	56.26	56.44	57.00	0.738	0.767
a *	13.40	15.46	16.56	0.841	0.319
b *	9.96	10.88	11.84	0.804	0.668

n = 18 (6 pigs per treatment); SEM: standard error of the mean; L *: Lightness; a *: Redness; b *: Yellowness. ^a,b^ Values (n = 6 per treatment) with no common superscript differ significantly (*p* ≤ 0.05).

**Table 9:** Effects of silage on meat microbial populations.

	Diets			SEM	*p*-Value
**Triceps Brachii Meat Microbes (Log CFU/g)**	**Silage-0%**	**Silage-5%**	**Silage-10%**		
Total Mesophilic count	4.99	3.94	4.83	0.23	0.14
*Campylobacter jejuni*	4.05 ^b^	3.51 ^ab^	3.09 ^a^	0.16	0.039
*Staphylococcus* spp.	2.78	2.37	2.04	0.24	0.72
*Staphylococcus aureus*	0.92	1.43	0.70	0.21	0.38
Sulfite-reducing *Clostridium*	1.01	0.85	1.15	0.18	0.81
**External abdominal oblique meat microbes (Log CFU/g)**					
Total Mesophilic count	5.59	5.34	4.95	0.23	0.55
*Campylobacter jejuni*	0.80	0.80	0.30	0.15	0.29
*Staphylococcus* spp.	2.97	3.07	3.05	0.15	0.97
*Staphylococcus aureus*	0.88	1.10	0.76	0.17	0.72
Sulfite-reducing *Clostridium*	1.55	1.47	1.28	1.49	0.58
*Escherichia coli*	1.04	1.15	1.46	0.17	0.61

n = 18 (6 pigs per treatment); SEM: Standard error of the mean; ^a,b^ Values with no common superscript differ significantly (*p* ≤ 0.05).

**Table 10:** Effects of silage addition on pig meat oxidative stability.

	Diets			SEM	*p*-Value
**Triceps Brachii Meat**	**Silage-0%**	**Silage-5%**	**Silage-10%**		
Total phenols (g/L)	1.51 ^a^	1.82 ^ab^	2.28 ^b^	0.116	0.013
TBARS (mg MDA/kg)	0.09	0.06	0.05	0.008	0.201
**External abdominal oblique meat**					
Total phenols (g/L)	1.90	2.29	2.24	0.112	0.332
TBARS (mg MDA/kg)	0.07	0.05	0.04	0.007	0.171

n = 18 (6 pigs per treatment); SEM: standard error of the mean; TBARS: Thiobarbituric acid reactive substances. MDA: Malondialdehyde; ^a,b^ Values with no common superscript differ significantly (*p* ≤ 0.05).

**Table 11:** Effect of silage supplementation on pig triceps brachii meat fatty acid composition.

	Diets			SEM	*p*-Value
**Triceps Brachii** **Meat FA (%)**	**Silage-0%**	**Silage-5%**	**Silage-10%**		
C8:0 (Caprylic)	0.01	0.01	0.01	-	-
C10:0 (Capric)	0.09	0.09	0.10	0.011	0.972
C12:0 (Lauric)	0.07	0.08	0.09	0.006	0.630
C14:0 (Myristic)	1.44	1.47	1.57	0.083	0.850
C14:1 (Myristoleic)	0.02	0.01	0.02	0.002	0.202
C15:0 (Pentadecanoic)	0.04	0.05	0.04	0.005	0.780
C16:0 (Palmitic)	26.59 ^xy^	25.64 ^x^	29.19 ^y^	0.693	0.071
C16:1 cis (Palmitoleic)	2.60	2.43	2.40	0.161	0.670
C17:0 (Heptadecanoic)	0.24	0.28	0.26	0.022	0.836
C17:1 (cis-10 Heptadecenoic cis)	0.16 ^x^	0.27 ^y^	0.25 ^xy^	0.023	0.055
C18:0 (Stearic)	11.83	11.78	12.31	0.247	0.693
C18:1n-9t (Elaidic)	0.19	0.09	0.19	0.039	0.543
C18:1 cis n-9 (Oleic)	37.65	40.05	38.20	0.821	0.522
C18:2n-6t (Linolelaidic)	0.03	0.03	0.02	0.003	0.702
C18:2 n-6c (Linoleic)	15.28	14.45	13.07	0.845	0.625
C18:3n-3 (a-Linolenic)	0.71	0.85	0.4	0.059	0.690
C18:3n-6 (γ-Linolenic)	0.08 ^y^	0.08 ^y^	0.03 ^x^	0.009	0.085
C20:0 (Arachidic)	0.05	0.09	0.10	0.010	0.178
C20:1 cis n-9 (cis-11 Eicosenoic)	0.33 ^x^	0.45 ^y^	0.42 ^xy^	0.026	0.097
C20:2 cis n-6 (cis-11,14-Eicosadienoic)	0.37	0.45	0.33	0.029	0.219
C20:3 cis n-3 (cis-11-14-17-Eicosatrienoate)	0.22	0.16	0.07	0.033	0.245
C20:4 cis n-6 (Arachidonic)	1.65 ^y^	0.93 ^xy^	0.46 ^x^	0.314	0.079
C20:5 cis n-3 (Cis-5,8,11,14,17-Eicosapentaenoic)	0.05 ^y^	0.03 ^xy^	0.01 ^x^	0.010	0.069
C21:0 (Henicosanoic)	0.03	0.04	0.02	0.004	0.138
C22:6 cis n-3 (cis-4,7,10,13,16,19-Docosahexaenoic)	0.08 ^y^	0.04 ^xy^	0.02 ^x^	0.120	0.057
C23:0 (Tricosanoic)	0.01	0.01	0.01	0.003	0.498
C24:1n-9 (Nervonic)	0.21 ^y^	0.13 ^xy^	0.05 ^x^	0.035	0.069
Σ SFA (Total Saturated FA)	40.40 ^a^	39.54 ^a^	43.60 ^b^	0.614	0.049
Σ MUFA (Total Monounsaturated FA)	40.95 ^a^	43.30 ^b^	41.48 ^a^	0.518	0.021
Σ PUFA (Total Polyunsaturated FA)	18.47 ^b^	17.02 ^b^	14.75 ^a^	0.535	0.023
Σ n-6 (Total omega-6 FA)	17.41	15.94	13.91	1.089	0.497
Σ n-3 (Total omega-3 FA)	1.06	1.07	0.84	0.071	0.405
Ratio n-6/n-3 FA	16.42	14.61	16.55	0.565	0.532
PUFA/SFA	0.45 ^y^	0.43 ^xy^	0.34 ^x^	0.029	0.06
h/H ^c^	1.99 ^xy^	2.09 ^y^	1.71 ^x^	0.093	0.097

n = 6 pigs per group. FA: Fatty acids; Σ SFA = (C10:0) + (C12:0) + (C14:0) + (C16:0) + (C17:0) + (C18:0); Σ MUFA = (C16:1 cis) + (C17:1 cis-10) + (C18:1 cis n9) + (C18:1 n7) + (C20:1 cis n9); Σ PUFA = (C18:2 n-6c) + (C18:4n-3) + (C20:2 cis n-6) + C20:3 cis n-6) + (C22:5 cis n-3) + (C20:3 cis n-3) + (C20:4 cis n-6) + (C20:5 cis n-3) + (C21:5 n-3) + (C22:6 cis n-3). ^a,b^ Values (n = 6 per treatment) with no common superscript differ significantly (*p* ≤ 0.05). ^x,y^ Values with no common superscript tend to (0.05 < *p* ≤ 0.10). ^c^ hypocholesterolemic/Hypercholesterolemic ratio = (cis-C18:1 + Σ PUFA)/(C12:0 + C14:0 + C16:0).

**Table 12:** Effect of silage supplementation on pig external abdominal meat fatty acid composition.

	Diets			SEM	*p*-Value
**External Abdominal Oblique** **Meat FA (%)**	**Silage-0%**	**Silage-5%**	**Silage-10%**		
C8:0 (Caprylic)	0.01	0.01	0.01	0.001	0.368
C10:0 (Capric)	0.13	0.10	0.12	0.016	0.394
C12:0 (Lauric)	0.10	0.09	0.11	0.005	0.422
C14:0 (Myristic)	1.75	1.81	1.97	0.060	0.327
C14:1 (Myristoleic)	0.02	0.02	0.02	0.002	0.264
C15:0 (Pentadecanoic)	0.04	0.04	0.04	0.003	0.702
C16:0 (Palmitic)	2.77	29.09	30.61	0.559	0.603
C16:1 cis (Palmitoleic)	2.95	2.27	2.72	0.167	0.301
C17:0 (Heptadecanoic)	0.19	0.25	0.23	0.013	0.241
C17:1 cis-10 (Heptadecenoic cis)	0.17 ^x^	0.24 ^y^	0.25 ^y^	0.018	0.059
C18:0 (Stearic)	11.73	12.85	11.79	0.516	0.676
C18:1n-9t (Elaidic)	0.07	0.21	0.23	0.032	0.066
C18:1 cis n-9 (Oleic)	41.09	38.91	38.24	1.222	0.673
C18:2n-6t (Linolelaidic)	0.01	0.03	0.02	0.002	0.125
C18:2 n-6c (Linoleic)	10.04	11.83	11.49	0.521	0.372
C18:3n-3 (a-Linolenic)	0.52	0.65	0.72	0.041	0.133
C18:3n-6 (γ-Linolenic)	0.02	0.04	0.03	0.003	0.296
C20:0 (Arachidic)	0.10	0.11	0.11	0.005	0.651
C20:1 cis n-9 (cis-11-Eicosenoic)	0.48	0.41	0.40	0.033	0.541
C20:2 cis n-6 (cis-11,14-Eicosadienoic)	0.32	0.34	0.33	0.009	0.637
C20:3 cis n-3 (cis-11-14-17-Eicosatrienoate)	0.06	0.07	0.06	0.033	0.202
C20:4 cis n-6 (Arachidonic)	0.31 ^x^	0.50 ^y^	0.36 ^xy^	0.038	0.097
C20:5 cis n-3 (Cis-5,8,11,14,17-Eicosapentaenoic)	0.01	0.01	0.01	-	-
C21:0 (Henicosanoic)	0.03	0.03	0.03	0.002	0.579
C22:6 cis n-3 (cis-4,7,10,13,16,19-Docosahexaenoic)	0.02	0.02	0.02	0.001	0.565
C24:1n-9 (Nervonic)	0.06	0.07	0.05	0.005	0.441
Σ SFA (Total Saturated FA)	43.85	44.38	45.02	0.490	0.653
Σ MUFA (Total Monounsaturated FA)	44.84 ^b^	42.13 ^ab^	41.91 ^a^	0.659	0.043
Σ PUFA (Total Polyunsaturated FA)	11.31	13.49	13.04	0.397	0.113
Σ n-6 (Total omega-6 FA)	10.70	12.74	12.23	0.544	0.328
Σ n-3 (Total omega-3 FA)	0.61	0.76	0.81	0.044	0.127
Ratio n-6/n-3 FA	17.54 ^b^	16.76 ^b^	15.09 ^a^	0.416	0.005
PUFA/SFA	0.25	0.30	0.29	0.021	0.944
h/H ^c^	1.65	1.69	1.56	0.062	0.838

n = 6 pigs per group. FA: Fatty acids; Σ SFA = (C10:0) + (C12:0) + (C14:0) + (C16:0) + (C17:0) + (C18:0); Σ MUFA = (C16:1 cis) + (C17:1 cis-10) + (C18:1 cis n9) + (C18:1 n7) + (C20:1 cis n9); Σ PUFA = (C18:2 n-6c) + (C18:4n-3) + (C20:2 cis n-6) + C20:3 cis n-6) + (C22:5 cis n-3) + (C20:3 cis n-3) + (C20:4 cis n-6) + (C20:5 cis n-3) + (C21:5 n-3) + (C22:6 cis n-3). ^a,b^ Values (n = 6 per treatment) with no common superscript differ significantly (*p* ≤ 0.05), ^x,y^ Values with no common superscript tend to (0.05 < *p* ≤ 0.10). ^c^ hypocholesterolemic/Hypercholesterolemic ratio = (cis-C18:1 + Σ PUFA)/(C12:0 + C14:0 + C16:0).

## Data Availability

The original contributions presented in this study are included in the article. Further inquiries can be directed to the corresponding author.
